# A217 A REVIEW OF MOBILE HEALTH APPLICATIONS FOR INDIVIDUALS LIVING WITH INFLAMMATORY BOWEL DISEASE USING MOBILE APPLICATION RATING SCALE (MARS)

**DOI:** 10.1093/jcag/gwac036.217

**Published:** 2023-03-07

**Authors:** N Jannati, S Salehinejad, E Kuenzig, J N Peña-Sánchez

**Affiliations:** 1 Community Health & Epidemiology, University of Saskatchewan, Saskatoon, Canada; 2 Health in Disasters and Emergencies Research Center, Institute for Futures Studies in Health, Kerman University of Medical Sciences, Kerman, Iran, Islamic Republic Of; 3 Child Health Evaluative Sciences, SickKids Research Institute; 4 SickKids Inflammatory Bowel Disease Centre, Division of Gastroenterology, Hepatology and Nutrition, The Hospital for Sick Children, Toronto; 5 Department of Community Health & Epidemiology, University of Saskatchewan, Saskatoon, Canada

## Abstract

**Background:**

Inflammatory bowel disease (IBD) is a chronic disorder that can be managed but not cured. The relapsing and remitting symptoms of IBD impact patients' quality of life and is associated with considerable healthcare costs. Different mobile health apps have been developed around the world for IBD and other chronic medical conditions. These apps can potentially decrease healthcare utilization and the burden of the disease.

**Purpose:**

We aimed to review IBD mobile health apps available in English and evaluate their quality and content.

**Method:**

We searched the Apple Store and Play Store in July 2022 using IBD-related keywords (e.g., “IBD,” “Crohn,” “Crohns,” “colitis,” “ulcerative colitis,” and “inflammatory bowel disease.”) to identify apps for iOS and Android devices, respectively. We included apps available in English, designed specifically for IBD, and free to download and use. Two researchers reviewed, rated, and evaluated the retrieved apps independently. The Mobile App Rating Scale (MARS) was used to rate the included apps. The MARS rates apps on a scale of 1 to 5 and includes an overall score as well as scores for engagement, functionality, information, aesthetics, and subject quality. A score ≥3 is deemed acceptable.

**Result(s):**

Search queries yielded 88 relevant apps, of which 38 met the inclusion criteria and were included in this review. The included apps for IBD were most tracking and monitoring symptoms of the disease (n=22). Two of the apps were affiliated with universities, and three of the apps were specifically developed for children. In addition, 11 apps were designed for IBD, regardless of disease subtype (Crohn’s disease [CD] or ulcerative colitis [UC]), and 27 apps were designed specifically for either UC or CD. The mean MARS score for IBD apps was 3.3 (SD=0.6), with more than half deemed acceptable. Apps scored highest on the functionality (mean=3.7, SD=0.6) and information (mean=3.6, SD=0.5) dimension of MARS and lowest on the engagement dimension (mean=3, SD=0.8).

**Image:**

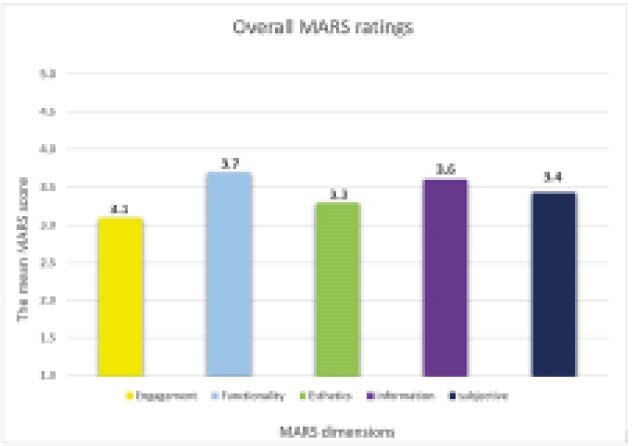

**Conclusion(s):**

Most IBD-related apps provide acceptable information and functionality but should improve their engagement to be welcomed by users. This review provides a roadmap for improving available apps and future app development for individuals with IBD. App developers in the field of IBD must ensure they create high-quality, engaging, esthetic and evidence-based apps.

**Please acknowledge all funding agencies by checking the applicable boxes below:**

None

**Disclosure of Interest:**

None Declared

